# Association between Gestational Weight Gain, Gestational Diabetes Risk, and Obstetric Outcomes: A Randomized Controlled Trial Post Hoc Analysis

**DOI:** 10.3390/nu10111568

**Published:** 2018-10-23

**Authors:** David Simmons, Roland Devlieger, Andre van Assche, Sander Galjaard, Rosa Corcoy, Juan M. Adelantado, Fidelma Dunne, Gernot Desoye, Alexandra Kautzky-Willer, Peter Damm, Elisabeth R. Mathiesen, Dorte M. Jensen, Lise Lotte T. Andersen, Annunziata Lapolla, Maria G. Dalfra, Alessandra Bertolotto, Ewa Wender-Ozegowska, Agnieszka Zawiejska, David Hill, Frank J. Snoek, Mireille N. M. van Poppel

**Affiliations:** 1 Institute of Metabolic Science, Addenbrooke’s Hospital, CB2 0QQ Cambridge, UK; alexandra.kautzky-willer@meduniwien.ac.at; 2 Macarthur Clinical School, Western Sydney University, Locked Bag 1797, Campbelltown, Sydney, NSW 2760, Australia; 3 Department of Development and Regeneration, KU Leuven, 3000 Leuven, Belgium; roland.devlieger@uz.kuleuven.ac.be (R.D.); andre.vanassche@kuleuven.be (A.v.A.); s.galjaard@erasmusmc.nl (S.G.); 4 Department of Obstetrics and Gynecology, University Hospitals Leuven, 3000 Leuven, Belgium; 5 Department of Obstetrics, Gynecology and Fertility, GasthuisZusters Antwerpen Sint-Augustinus, 2610 Wilrijk, Belgium; 6 Department of Obstetrics and Gynaecology, Division of Obstetrics and Prenatal Medicine, Erasmus MC, University Medical Centre, 3015 GD Rotterdam, The Netherlands; 7 CIBER Bioengineering, Biomaterials and Nanomedicine, Instituto de Salud Carlos III, Zaragoza, 50018 Spain; RCorcoy@santpau.cat; 8 Institut de Recerca de l’Hospital de la Santa Creu i Sant Pau, 08025 Barcelona, Spain; JAdelantado@santpau.cat; 9 Galway Diabetes Research Centre (GDRC) and National University of Ireland, H91 CF50 Galway, Ireland; fidelma.dunne@nuigalway.ie; 10 Department of Obstetrics and Gynecology, Medizinische Universitaet Graz, A-8036 Graz, Austria; gernot.desoye@medunigraz.at; 11 Center for Pregnant Women with Diabetes, Departments of Endocrinology and Obstetrics, Rigshospitalet and The Clinical Institute of Medicine, Faculty of Health and Medical Sciences, University of Copenhagen, DK-1165 Copenhagen, Denmark; peter.damm@regionh.dk (P.D.); elisabeth.mathiesen@rh.regionh.dk (E.R.M.); 12 Department of Endocrinology, Odense University Hospital, DK-5000 Odense, Denmark; Dorte.Moeller.Jensen@ouh.regionsyddanmark.dk; 13 Department of Gynaecology and Obstetrics, Odense University Hospital, DK-5000 Odense, Denmark; 14 Department of Clinical Research, Faculty of Health Sciences, University of Southern Denmark, DK-5230 Odense, Denmark; lise.lotte.andersen@rsyd.dk; 15 Department of Medical and Surgical Sciences. Università degli Studi di Padova, 35100 Padua, Italy; annunziata.lapolla@unipd.it (A.L.); mariagrazia.dalfra@sanita.padova.it (M.G.D.); 16 Department of Clinical and Experimental Medicine, University of Pisa, 56126 Pisa, Italy; alessandrabertolotto1959@yahoo.it (A.B.); 17 Department of Reproduction, Poznan University of Medical Sciences, 61-701 Poznan, Poland; ewaoz@post.pl (E.W.-O.); agazaw@post.pl (A.Z.); 18 Recherche en Santé Lawson SA, 9552 St. Gallen, Switzerland; David.Hill@LawsonResearch.Com; 19 Department of Public and Occupational Health, Amsterdam Public Health Research Institute, Amsterdam UMC, Vrije Universiteit Amsterdam, Van der Boechorststraat 7, NL-1081 BT Amsterdam, The Netherlands.; fj.snoek@vumc.nl (F.J.S.); Mireille.van-poppel@uni-graz.at (M.N.M.v.P.); 20 Institute of Sports Sciences, University of Graz, A-8010 Graz, Austria

**Keywords:** gestational diabetes mellitus, pregnancy, lifestyle intervention, randomised controlled trial, healthy eating, physical activity, overweight, motivational interviewing, prevention

## Abstract

Excess gestational weight gain (GWG) is associated with the development of gestational diabetes mellitus (GDM). Lifestyle trials have not achieved much GWG limitation, and have largely failed to prevent GDM. We compared the effect of substantial GWG limitation on maternal GDM risk. Pregnant women with a body mass index (BMI) ≥29 kg/m^2^ <20 weeks gestation without GDM (*n* = 436) were randomized, in a multicenter trial, to usual care (UC), healthy eating (HE), physical activity (PA), or HE and PA lifestyle interventions. GWG over the median was associated with higher homeostasis model assessment insulin resistance (HOMA-IR) and insulin secretion (Stumvoll phases 1 and 2), a higher fasting plasma glucose (FPG) at 24–28 weeks (4.66 ± 0.43 vs. 4.61 ± 0.40 mmol/L, *p* < 0.01), and a higher rate of caesarean section (38% vs. 27% *p* < 0.05). The GWG over the median at 35–37 weeks was associated with a higher rate of macrosomia (25% vs. 16%, *p* < 0.05). A post hoc comparison among women from the five sites with a GWG difference >3 kg showed no significance difference in glycaemia or insulin resistance between HE and PA, and UC. We conclude that preventing even substantial increases in GWG after the first trimester has little effect on maternal glycaemia. We recommend randomized controlled trials of effective lifestyle interventions, starting in or before the first trimester.

## 1. Introduction

The risk of gestational diabetes mellitus (GDM) increases with both obesity and gestational weight gain (GWG) [1] GDM, excess GWG, and overweight/obesity are all independently associated with an increased risk of macrosomia, operative delivery, and other adverse perinatal outcomes, including shoulder dystocia [1]. If increased GWG is causally related to an increase in GDM incidence, then limiting GWG should reduce the incidence of GDM. However, the results from randomized controlled trials (RCTs) for the prevention of GDM through lifestyle have been mixed [2]. The mean difference in GWG between the intervention and control groups has ranged between −9.07 kg and +3.5 kg in a meta-analysis in 2012, with a mean of −2.21 kg and no significant reduction in GDM rates [3]. A recent meta-analysis of the individual participant data from randomized trials estimated a GWG reduction of 0.7 kg overall, with no overall reduction in GDM (unless data from studies without individual data were included) [4]. A GDM reduction was found with interventions involving physical activity alone (PA) [4]. The most recent Cochrane review (2017) showed no reduction in GDM or in adverse obstetric outcomes with lifestyle interventions [5]. However, another recent meta-analysis showed a reduction in GDM if intervention commenced in the first, but not the second trimester [6]. Moreover, prevention occurred in some lifestyle intervention studies with minimal GWG difference (e.g., The Finnish Gestational Diabetes Prevention Study (RADIEL), where women with previous GDM and/or obesity were studied) [7]. More information is therefore needed on the relationship between GWG, the development of GDM, and obstetric outcomes.

The Vitamin D and Lifestyle Intervention for GDM Prevention (DALI) [8,9,10] is a European multicenter RCT that tested different approaches for the reduction of GDM risk. The study was unique in that it had the following two limbs: (1) the DALI Lifestyle Study, which compared healthy eating (HE), PA, and mixed (HE and PA) with a control group, and (2) the DALI Vitamin D study that compared vitamin D supplementation with and without an HE and PA intervention. The main lifestyle RCT (the DALI Lifestyle Study) found the HE and PA intervention was associated with the least GWG, but there was no significant reduction in the fasting glucose, GDM incidence, or insulin sensitivity (as measured with the homeostasis model assessment insulin resistance (HOMA-IR) [10].

We hypothesized that the greater the GWG limitation, the greater the difference in GDM rates, fasting glucose, HOMA, and adverse obstetric outcomes between controls and intervention subjects. We tested this by initially comparing women above and below the median GWG, independent of intervention. As randomization in the DALI Lifestyle Study was stratified by site, we have gone on to treat each site within this study as a separate RCT, and tested whether the GDM risk was reduced in the five sites with the greatest GWG limitation, using the HE and PA intervention—the only intervention able to achieve GWG limitation.

## 2. Materials and Methods

### 2.1. Overall Study Design

The methods used in this study have been described previously [8,9,10]. Briefly, the DALI Lifestyle Study is an RCT **Trial registration:** ISRCTN70595832 that compared three different lifestyle approaches that could prevent GDM across the following ten European centers: Cambridge, (United Kingdon: coordinating center), Amsterdam (Netherlands), Leuven (Belgium), Barcelona (Spain), Galway (Ireland), Pisa/Padova (two sites; Italy), Vienna (Austria), Poznan (Poland), Copenhagen, and Odense (Denmark). The primary outcomes were changes in the GWG, fasting glucose, and HOMA-IR. The target GWG was 5 kg, which is at the lower end of the Institute of Medicine’s recommendations [11].

The women were randomized, and were stratified by site to usual care (UC), HE, PA, or HE and PA. The intervention has been described in detail previously [8,10]. After randomization, the women were assigned a lifestyle coach who provided five face-to-face, and up to four telephone coaching sessions, based on the principles of motivational interviewing. The coaches received standardized training and an intervention toolkit (including, e.g., a pedometer in the PA intervention) tailored to their culture/language. The coaching involved the discussion of seven HE and/or five PA “messages”, and a GWG <5 kg was targeted [10]. The HE intervention promoted a food-based, lower simple and complex carbohydrate, lower fat, higher fiber, and higher protein diet, including a focus on portion size, and therefore a more limited intake of total calories. The PA intervention promoted both aerobic and resistance physical activity. At least four face-to-face coaching sessions were expected to take place before the second measurement session (24–28 weeks), and the intervention was completed after 35 weeks of gestation. The HE intervention was associated with the self-reported reduced carbohydrate intake, reduced portion size, increased vegetable intake, and reduced sugary drinks intake, while the PA intervention was associated with a reduced sedentary time and increased moderate or vigorous activity [10]. The combined intervention was the only intervention associated with a significant reduction in GWG.

The participants were pregnant women aged ≥18 years, before 20 weeks gestation, with a pre-pregnancy body mass index (BMI) ≥29 kg/m^2^, and were recruited between January 2012 and February 2014. All of the women underwent an oral glucose tolerance test (OGTT), and those with GDM (International Association Diabetes Pregnancy Study Group (IADPSG)/World Health Organisation (WHO) 2013 criteria of fasting venous plasma glucose ≥5.1 mmol/L and/or one hour of glucose ≥10 mmol/L and/or two hours of glucose ≥8.5 mmol/L) [12] were excluded from the study. Other exclusion criteria are reported elsewhere [8]. All of the women gave signed informed consent. The study was approved by the relevant ethical committees and was registered as an RCT (ISRCTN70595832). The assessments were made by the research midwife/nurse at four antenatal time points—before 20 weeks (baseline), between 24–28 weeks (visit 2), and between 35–37 weeks (visit 3) gestation. Maternal gestational weight gain (GWG) was defined as the weight difference between the self-reported pre-pregnancy and the DALI antenatal measurement. Randomization was stratified by site, and the randomization method, and interventions have been described in detail previously [8]. Those involved in taking the measurements were kept blinded to the intervention.

Information on the demographics, pre-pregnancy weight, smoking, alcohol consumption, past/current medical and obstetric history, and medication use was gathered using questionnaires. The data from the medical records were obtained regarding the co-morbidities, obstetric and perinatal outcomes, and birth weight. The women attended the three assessments while fasting, and undertook a standardized, sitting, 75 g OGTT, with blood samples taken at 0, 60, and 120 min after glucose ingestion. The women completed the questionnaire and anthropometric measurements between the blood tests. The local laboratories were used to rapidly obtain the OGTT results so as to assess their eligibility for the study, and to support referral for clinical care where needed. The blood samples were centrifuged and separated from the serum and plasma aliquots (1000 µL or 250 µL), placed in microrack tubes, and stored at −20 or −80 °C, until further analysis, in the central trial laboratory in Graz, Austria, which was certified according to ISO 9001 standards.

The laboratory glucose and insulin analytical methods have been described previously [8,9,10]. The homeostatic model assessment-insulin resistance (HOMA-IR) was calculated as (glucose × insulin)/22.5 and the homeostatic model assessment-insulin secretion (HOMA-IS) (B) was calculated using the following formula of 20 × fasting insulin (μIU/mL)/fasting glucose (mmol/mL) − 3.5 [13]. The Stumvoll first and second phase indices are the surrogates of early and late insulin secretion, and were calculated as 1194 + 4.724 × Ins0 − 117.0 × Gluc60 + 1.414 × Ins60 for the Stumvoll first phase, and 295 + 0.349 × Ins60 − 25.72 × Gluc60 + 1.107 × Ins0 for the Stumvoll second phase, as described earlier by Stumvoll et al. [14,15]

The height was measured at baseline with a stadiometer (SECA 206, SECA, Birmingham, UK; Leicester Height Measure), and the average value of the two measurements was used. The women were weighed on calibrated electronic scales (SECA 888; SECA 877, SECA, Birmingham, UK) without shoes and wearing light clothes, to the nearest 0.1 kg; the average value of the two measurements was used. 

### 2.2. Statistics

The trial data were entered into a bespoke web-based electronic database using the Microsoft.Net development environment. The analyses were performed in SPSS22. The trial data were analyzed according to the intention-to-treat principle. Two-sided *p* < 0.05 was taken as significant. The discrete variables were described as crude %, with comparisons adjusted for sites using logistic regression, and were reported with 95% confidence intervals. The continuous variables were compared using generalized linear modelling, again adjusting for sites, with the mean ± SEM shown. The comparisons were also adjusted for the other covariates, as described. The following two analyses were undertaken in this post hoc study: The first was to compare the characteristics of the women above and below the median GWG at each gestation. This comparison was undertaken rather than above and below the lower limit of the Institute of Medicine (IOM) [11] targets so as to maximize the numbers in both groups, and only a minority of women achieved the 5 kg limitation. The second analysis used the RCT framework to compare the impact of a substantial GWG limitation on metabolic the and obstetric outcomes. A substantial GWG limitation was defined as >50% higher than the mean DALI Lifestyle Trial GWG difference between the UC and intervention groups (i.e., >3 kg difference). This was only achieved with the HE and PA intervention in five sites, and hence the post hoc comparison was only undertaken between UC, and HE and PA in these five sites.

## 3. Results

The gestational age on entry ranged from 8 to 19^+6^ weeks. The median GWG between pre-pregnancy and 24–28 weeks was 5.5 (IQR 2.3–9.1) kg, and at 35–37 weeks it was 9.5 (IQR 5.3–14.3) kg. Table 1 shows the baseline data for those above and below the median GWG at baseline, 24–28 weeks overall, and at 35–37 weeks, excluding those developing GDM by 24–28 weeks gestation. The women with the greatest GWG commenced at a lower weight and body mass index (BMI), and were more likely to be smokers and nulliparous (35–37 weeks group only). Those with the greatest GWG at 24–28 weeks had a lower fasting glucose, while those with the greatest GWG at 35–37 weeks (excluding those with GDM by 24–28 weeks) had lower fasting, and 1 h and 2 h glucose concentrations at baseline.

Table 2 shows that the HOMA-IR, Stumvoll phase 1, and Stumvoll phase 2 were significantly higher among those with the greatest GWG by 24–28 weeks and 35–37 weeks. HOMA-IS was also statistically higher in those with the greatest GWG at 24–28 weeks. The fasting glucose was higher in those with the greatest GWG at 24–28 weeks and 35–37 weeks. The women with the greatest GWG by 35–37 weeks (but not by 24–28 weeks) had significantly larger babies. There were no other significant obstetric differences. There were no differences in the gender of the babies between groups by GWG.

**Figure 1:**
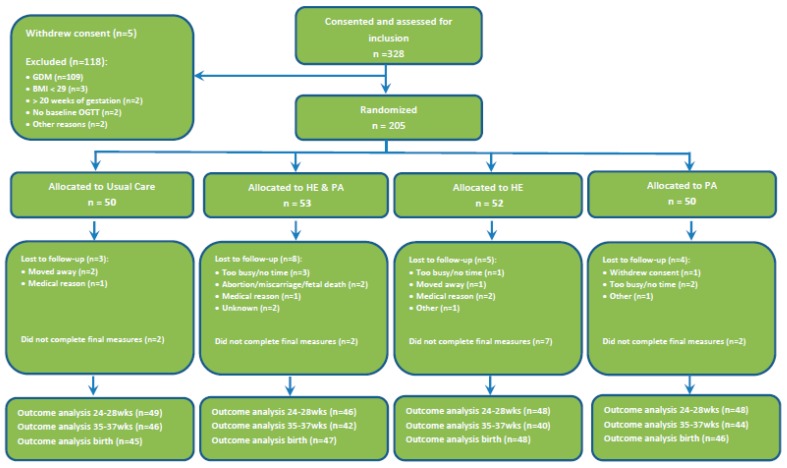
Recruitment flowchart for five sites achieving the greatest gestational weight gain limitation.

Figure 1 shows the flow of the participants in the five sites with a high GWG difference throughout the post hoc analysis. The numbers that were randomized were comparable. Table 3 compares the maternal characteristics and the pregnancy outcome measures between usual care (UC), and HE and PA. The women in the HE and PA group had 2.6 kg less GWG at 24–28 weeks and 4.3 kg at 35–37 weeks compared with the UC group. The glucose levels, measures of insulin resistance and secretion, and GDM rates were not significantly different. The combined lifestyle intervention was associated with a significantly lower large for gestational age (LGA) rate.

## 4. Discussion

We have shown that GWG above the median is indeed associated with increased fasting glucose, insulin resistance, and insulin secretion. However, there was no increased risk of GDM. In a post hoc analysis in the five sites with an average 4.3 kg GWG reduction in the HE and PA intervention by 35–37 weeks, there were similar rates of GDM and no difference in the glycaemia, HOMA-IR, or measures of insulin secretion. A GWG above the median in the first and third trimesters was also associated with more caesarean sections and greater macrosomia/LGA, respectively. The HE and PA intervention with its associated GWG limitation showed a significant reduction in LGA—something not shown in previous meta-analyses [4,5] with their lower GWG limitation.

No other large study has achieved this degree of GWG limitation [3]. We believe that this post hoc study clearly demonstrates that GDM cannot be prevented in overweight/obese women with lifestyle intervention initiated in the second trimester, even with substantial GWG limitation. Previous studies have failed to conclusively demonstrate this lack of effect, because of the limited effect of their lifestyle interventions on GWG [4]. There are two key questions that arise, as follows: firstly, why is it that there was no effect on the risk of GDM, and, secondly, what other strategies might be effective?

There are several theories behind why lifestyle change, even when resulting in a significant GWG limitation, is insufficient to prevent GDM in overweight/obese women. The first is that there is simply insufficient time between commencing the intervention and the OGTT at 24 weeks of gestation for the intervention to be effective. Certainly, some subjects in the DALI study only experienced 4–8 weeks of the intervention. A further possibility is that the reduction in insulin resistance achievable with lifestyle is insufficient. While the mean insulin resistance was reduced by 10–15%, this was not statistically significant and was unlikely to be physiologically meaningful, given the fact that the overall increase in insulin resistance during pregnancy is two- to three-fold [16]. A parallel situation occurs with the use of metformin for the prevention of GDM. While metformin prevents type 2 diabetes over time [17], no reduction in the development of GDM was seen in either the Effect of Metformin on Maternal and Fetal Outcomes in Obese Pregnant Women (EMPOWaR) or in the Metformin in Obese Non-Diabetic Pregnant Women (MOP) RCTs of metformin for the prevention of GDM [18,19], although MOP did show a reduction in insulin resistance [20]. However, one would expect that the intervention would have been effective in reducing GDM in a proportion of women, but this was not seen.

The relationship between insulin resistance and insulin secretion follows a clear hyperbola [21], and as long as the insulin secretory capacity is sufficient, the glucose homeostasis would be predicted to remain steady, and prevent the development of GDM. An increase in insulin resistance was shown in our comparison of women with GWG above and below the median, and insulin secretion was also greater, suggesting a compensatory increase. In women with previous GDM, the development of type 2 diabetes outside of pregnancy was prevented with troglitazone, a thiazolidinedione that also reduces insulin resistance [22]. The proposed mechanism was that the reduced insulin resistance reduced the insulin secretion, thereby preventing/delaying the onset of beta cell exhaustion, and the drop off from the set insulin resistance–insulin secretion hyperbola. While an estimated increase in insulin sensitivity of 88% was seen within three months [23], prevention occurred over a median of 30 months, much longer than the time available during pregnancy, and without the need to adapt to rapidly changing insulin requirements. One possibility is that the trajectory of insulin secretion by the beta cell is already established by the second trimester (i.e., some form of “programming” has occurred in the first trimester) [24].

The possibility of maternal metabolic trajectory setting in the first trimester as an explanation for the failure to prevent GDM in DALI is supported by the recent meta-analysis by Song [6], and another recent study introducing physical activity in the first trimester, which resulted in a substantial reduction in GDM [25]. The possibility of trajectory setting is also supported by the study by Hedderson et al. [26], where women with GWG in the highest tertile in the first trimester had an increased risk of GDM, but a similar relationship between GWG and GDM risk was not found in the second trimester. However, the mechanism for such a trajectory setting is unclear. 

If the trajectory is set in the first trimester, then there are two possible approaches to prevent GDM through standard (i.e., through some non-tested strategy) lifestyle changes among obese women. The first is for interventions to commence early in the first trimester, as suggested by Song [6]. However, for many women, the first contact with the health service is near the end of (or after) the first trimester, and an intervention may be challenging to commence. Nevertheless, this is certainly an aspect that warrants an RCT commencing between, for example, 4–8 weeks’ gestation. There is no evidence that such an RCT would increase spontaneous miscarriage. This may either involve a community wide intervention or personalized strategies, and may be before the first pregnancy or between pregnancies. One possible recommendation would be for obese women to plan their pregnancies in a comparable manner to women with pre-existing diabetes.

We have also shown that a substantial GWG limitation (mean 4.3 kg vs. usual care) is associated with reduced rates of LGA. This was shown in both the comparison of GWG above and below the median at 35–37 weeks, and with the RCT of usual care vs. the combined HE and PA intervention. Few studies have shown such an effect in pregnancy. The LIMIT RCT found a reduction after a post hoc analysis [27], but overall no beneficial effect has been shown [5]. The post hoc analysis undertaken here suggests that lifestyle interventions can reduce LGA rates, but that the degree of GWG reduction needs to be sufficient. There was a non-significantly higher rate of small for gestational age (SGA), and larger RCTs are needed to assess whether this degree of GWG limitation can restrict growth excessively.

This study has a number of strengths. It is one of the larger RCTs on lifestyle to prevent GDM, and it compared three different interventions. DALI was across nine European countries, with the participants encompassing a range of lifestyles and cultures, making it more widely representative than studies within a single site or country. The intervention was clearly effective, and being within site randomization allowed for the post hoc analysis. On the other hand, there are some shortcomings. In retrospect, the number of patients successfully recruited was sub-optimal to answer definitively important questions regarding a reduction in fasting glucose with lifestyle change and its relationship to the development of GDM. Nevertheless, the sample size provided 82% power to detect a 0.2 mmol/L difference in the fasting glucose, representing a clinically meaningful difference in the context of pregnancy. This is a post hoc analysis, with all of the statistical limitations that result from such an analysis. On the other hand, achieving GWG reduction goals is remarkably difficult, and to only select sites with an achieved GWG above the median is a novel way to test this within an RCT framework. We have not compared above and below the IOM lower target (5 kg), as only a limited number of women achieved this goal. Women coming into an RCT are more motivated than most women, and some women in the control arm appeared to be motivated to improve their lifestyle as a result of participation, thus reducing the differences between the intervention and control group in some sites. A further issue is the exclusion of women with GDM at baseline. DALI is one of the few to include this design feature, and while this allows a test of the impact of GWG limitation on the incidence of GDM, those with hyperglycemia at baseline may have had greater benefit from the intervention [28,29].

## 5. Conclusions

In conclusion, we have shown in a lifestyle intervention study RCT cohort that GWG is associated with an increase in insulin resistance and glycaemia and some worse obstetric outcomes, however, a post hoc analysis of RCT sites with the greatest GWG limitation showed no reduction in GDM or its risk, but a reduction in LGA. We conclude that lifestyle intervention in the second trimester is too late for GDM prevention, and more RCTs are needed in the first trimester, which includes sub-studies, so as to understand the mechanisms behind a putative “locking in” of the metabolic trajectory.

## Figures and Tables

**Table 1:** Maternal baseline characteristics in the Vitamin D and Lifestyle Intervention for Gestational Diabetes Mellitus (GDM) Prevention (DALI) participants, above and below median gestational weight gain from pre-pregnancy to baseline, to 24–28 weeks, and to 35–37 weeks gestation. The latter excludes women with GDM at 24–28 weeks.

Gestational Weight Gain Group	<1.80 kg at Baseline	≥1.80 kg at Baseline	<5.65 kg to 24–28 Weeks	≥5.65 kg to 24–28 Weeks	<9.5 kg to 35–37 Weeks †	≥9.5 kg to 35–37 Weeks †
	*n* = 215	*n* = 216	*n* = 203	*n* = 201	*n* = 158	*n* = 159
Age (years)	31.5 ± 5.3	32.4 ± 5.4	32.0 ± 5.2	32.1 ± 5.4	32.7 ± 5.1	31.7±5.4
Pre-pregnancy weight (kg)	**94.1 ± 14.0**	91.4 ± 12.0 *	**94.9 ± 14.0**	90.5 ± 11.9 ***	**94.7 ± 13.9**	90.8 ± 11.4 **
Pre-pregnancy BMI (kg/m^2^)	**34.2 ± 4.2**	33.3 ± 3.6 **	**34.6 ± 4.2**	33.0 ± 3.5 ***	**34.4 ± 4.1**	33.0 ± 3.5 **
European descent	184/215 (86%)	189/216 (88%)	177/203 (87%)	176/201 (88%)	137/158 (87%)	142/159 (89%)
Nullipara	109/215 (51%)	106/216 (49%)	100/203 (49%)	102/201 (51%)	**69/158 (44%)**	84/159 (53%) *
Smokers	26/215 (12%)	41/216 (19%)	**19/203 (9%)**	42/201 (21%) **	**14/158 (9%)**	29/159 (18%) *
First degree relative with diabetes	52/215 (24%)	48/216 (22%)	48/2103 (24%)	41/201 (20%)	33/158 (21%)	38/159 (24%)
Previous GDM	7/136 (5%)	10/137 (7%)	8/133 (6%)	5/120 (4%)	4/112 (4%)	6/91(7%)
Fasting glucose (mmol/L)	4.6 ± 0.4	4.6 ± 0.4	4.7 ± 0.4	4.6 ± 0.4	**4.7 ± 0.3**	4.6 ± 0.4 **
1-h glucose (mmol/L)	6.9 ± 1.4	6.7 ± 1.3	6.9 ± 1.4	6.7 ± 1.4	**6.9 ± 1.3**	6.5 ± 1.3 **
2-h glucose (mmol/L)	5.9 ± 1.2	5.8 ± 1.0	5.9 ± 1.1	5.8 ± 1.1	**5.9 ± 1.0**	5.7 ± 1.1 *
HOMA-IR (IQR)	2.5 (2.0, 3.4)	2.8 (2.0, 3.7)	2.7 (2.0, 3.6)	2.6 (2.0, 2.4)	2.7 (2.1, 3.4)	2.5 (1.9, 3.4)
HOMA insulin secretion (IQR)	217 (170, 312)	256 (180, 363)	220 (166, 324)	245 (181, 346)	215 (158, 295)	251 (180, 358)
Stumvoll phase 1 (IQR)	1590 (1221, 2067)	1521 (1255, 2045)	1560 (1219, 2083)	1502 (1258, 2016)	1595 (1224, 2099)	1507 (1261, 2042)
Stumvoll phase 2 (IQR)	409 (318, 532)	388 (327, 520)	408 (319, 533)	386 (326, 519)	410 (319, 534)	388 (327, 519)

For continuous outcomes, the differences between the groups were tested using multilevel regression models (country and individual as levels), and were adjusted for gestational age at the outcome measurement. For the dichotomous outcomes, logistic regression models were performed, and were adjusted for country and gestational age at the outcome measurement. * *p* < 0.05; ** *p* < 0.01; *** *p* < 0.001. † Excluding women with GDM at 24–28 weeks, based on local glucose values. BMI—body mass index; HOMA-IR—homeostasis model assessment insulin resistance. Numbers in bold highlight statistically significant comparisons.

**Table 2:** Metabolic status at 24–28 weeks, 35–37 weeks, and birth outcomes in DALI participants, according to gestational weight gain from pre-pregnancy.

Gestational Weight Gain Group	<1.80 kg at Baseline	≥1.80 kg at Baseline	<5.65 kg to 24–28 Weeks	≥5.65 kg to 24–28 Weeks	<9.5 kg to 35–37 Weeks †	≥9.5 kg to 35–37 Weeks †
	*n* = 215	*n* = 216	*n* = 203	*n* = 201	*n* = 156	*n* = 158
24–28 weeks						
Weight (kg)	**96.5 ± 12.4**	100.7 ± 12.8 **	**96.7±12.7**	100.4±12.7*	**96.7 ± 12.7**	100.2 ± 12.6 *
Gestational weight gain (kg) #	**2.3 ± 3.9**	9.3 ± 4.3 ***	**1.7±3.3**	9.9±3.8***	**2.0 ± 3.5**	9.4 ± 4.3 ***
Fasting blood glucose (BG) (mmol/l) #	**4.62 ± 0.40**	4.66 ± 0.42 *	**4.61±0.40**	4.66±0.43**	**4.58 ± 0.36**	4.57 ± 0.37 *
1-h glucose (mmol/l) #	7.73 ± 1.58	7.81 ± 1.66	7.74±1.59	7.83±1.68	7.52 ± 1.29	7.39 ± 1.49
2-h glucose (mmol/l) #	6.34 ± 1.23	6.21 ± 1.24	6.33±1.26	6.22±1.19	6.18 ± 1.02	5.98 ± 1.13
HOMA-IR #	**2.88 (2.17, 3.83)**	3.13 (2.39, 4.45) *	**2.80 (2.07, 3.76)**	3.15 (2.46, 4.47) ***	**2.84 (2.17, 3.69)**	3.03 (2.26, 4.29) ***
HOMA insulin secretion #	256 (190, 366)	285 (200, 393)	**264 (189, 345)**	283 (204, 407) *	269 (193, 343)	300 (208, 420)
Stumvoll phase 1 #	**1757 (1292, 2308)**	1888 (1404, 2347) *	**1675 (1276, 2256)**	1919 (1403, 2378) ***	**1612 (1287, 2255)**	1929 (1494, 2366) ***
Stumvoll phase 2 #	**454 (337, 588)**	484 (365, 598) *	**433 (335, 576)**	496 (368, 607) ***	**418 (336, 576)**	495 (380, 604) ***
35–37 weeks						
Weight (kg)†	**100.3 ± 12.3**	104.9 ± 13.0 **	**100.1 ± 12.6**	105.2 ± 12.8 **	**99.4 ± 12.8**	105.7 ± 12.3 ***
Gestational weight gain (kg) #†	**6.2 ± 5.3**	13.4 ± 5.7 ***	**5.4 ± 4.5**	14.5 ± 4.9 ***	**4.7 ± 3.7**	14.9 ± 4.4 ***
Fasting BG (mmol/l) #$	4.57 ± 0.45	4.60 ± 0.51	**4.55 ± 0.45**	4.61 ± 0.51 *	**4.49 ± 0.43**	4.53 ± 0.43 **
1-h glucose (mmol/l) #$	8.20 ± 1.61	8.46 ± 1.57	**8.21 ± 1.54**	8.45 ± 1.67 *	**8.01 ± 1.37**	8.17 ± 1.47 *
2-h glucose (mmol/l) #$	6.74 ± 1.28	6.58 ± 1.21	**6.82 ± 1.31**	6.51 ± 1.14 *	6.63 ± 1.21	6.43 ± 1.09
HOMA-IR #$	3.11 (2.35, 4.46)	3.38 (2.55, 4.60)	**2.96 (2,25, 4.32)**	3.45 (2.62, 4.76) **	**2.66 (2.21, 4.04)**	3.47 (2.70, 4.74) ***
HOMA insulin secretion #$	345 (234, 565)	354 (250, 483)	346 (227, 478)	351 (252, 531)	324 (217, 445)	367 (256, 531)
Stumvoll phase 1 #$	2469 (1722, 3174)	2518 (1898, 3117)	**2403 (1723, 3124)**	2561 (1917, 3200) **	**2383 (1732, 3026)**	2684 (2162, 3354) ***
Stumvoll phase 2 #$	629 (441, 801)	639 (497, 786)	**618 (450, 790)**	652 (497, 810) **	**608 (452, 766)**	684 (554, 841) ***
Birth	*N* = 198	*N* = 195	*N* = 194	*N* = 194	*N* = 154	*N* = 158
Gestation at birth (weeks)	39.5 ± 2.6	39.6 ± 1.7	39.8 ± 1.6	39.5 ± 1.7	39.8 ± 1.4	39.8 ± 1.3
Gender (male)	102/198 (52%)	94/195 (48%)	101/194 (52%)	94/194 (49%)	84/154 (55%)	74/158 (47%)
Birthweight	3490 ± 538	3479 ± 557	3457 ± 541	3505 ± 551	3477 ± 503	3602 ± 515
Birthweight ≥4 (kg)	37/195 (19%)	34/195 (17%)	33/192 (17%)	35/193 (18%)	**25/153 (16%)**	39/157 (25%) *
Birthweight <2.5 (kg)	8/195 (4%)	8/195 (4%)	8/192 (4%)	8/193 (4%)	5/153 (3%)	2/157 (1%)
Large for Gestational Age	26/187 (14%)	25/186 (13%)	23/186 (12%)	26/182 (14%)	**13/150 (9%)**	31/150 (21%) **
Small for Gestational Age	12/186 (7%)	16/186 (9%)	15/186 (8%)	13/182 (7%)	10/150 (7%)	9/150 (6%)
Preterm birth	8/194 (4%)	14/195 (7%)	8/192 (4%)	14/192 (7%)	2/153 (1%)	4/157 (3%)
Induction of labor or planned caesarean section	70/188 (37%)	82/187 (44%)	76/186 (41%)	75/184 (41%)	61/151 40%	60/148 41%
Caesarean section	**51/190 (27%)**	73/190 (38%) *	58/188 (31%)	65/187 (35%)	45/153 (29%)	53/151 (35%)
Pre-eclampsia	4/181 (2%)	10/187 (5%)	7/181 (4%)	8/183 (4%)	4/147 (3%)	5/148 (3%)
Neonatal Intensive Care Unit admission	19/170 (11%)	18/185 (10%)	19/173 (11%)	19/179 (11%)	14/145 (10%)	14/144 (10%)
GDM total	71/183 (39%)	61/175 (35%)	68/185 (37%)	63/175 (36%)	40/153 (26%)	45/155 (29%)

For the continuous outcomes, the differences between the groups were tested with multilevel regression models (country and individual as levels), and adjusted for the gestational age at the outcome measurement. For the dichotomous outcomes, logistic regression models were performed, and were adjusted for country and gestational age at outcome measurement. The data are adjusted for the pre-pregnancy BMI and fasting glucose at baseline. * *p* < 0.05; ** *p* < 0.01; *** *p* < 0.001. #—regression models were additionally adjusted either for value at baseline or for pre-pregnancy BMI when GWG was the outcome. $—value of 24–28 weeks carried forward to 35–37 weeks when GDM was diagnosed at 24–28 weeks. †—excluding women with GDM at 24–28 weeks, based on local glucose values. Numbers in bold highlight statistically significant comparisons.

**Table 3:** Maternal and neonatal characteristics of women at the five DALI sites with maternal gestational weight gain >3 kg difference at 35–37 weeks between healthy eating (HE), and physical activity intervention (HE and PA) and usual care (UC).

Baseline	UC	HE + PA
	*n* = 50	*n* = 53
Age (years)	32.1 ± 6.0	31.9 ± 5.0
Pre-pregnancy weight (kg)	94.7 ± 11.4	94.7 ± 13.6
Pre- pregnancy BMI (kg/m^2^)	33.6 ± 3.3	34.8 ± 4.1
Fasting (F) BG (mmol/L)	4.69 ± 0.33	4.64 ± 0.36
1-h BG (mmol/L)	6.80 ± 1.36	6.84 ± 1.23
2-h BG (mmol/L)	5.69 ± 1.25	5.72 ± 0.99
HOMA-IR	2.62 (1.94, 4.30)	2.49 (2.19, 2.82)
HOMA insulin secretion	229 (142, 365)	215 (168, 313)
Stumvoll phase 1	1498 (1211, 2144)	1489 (1236, 1948)
Stumvoll phase 2	379 (313, 554)	382 (322, 501)
European descent	45/50 90.0%	47/53 88.7%
Nullipara	27/50 46.2%	27/53 49.1%
Smokers	6/50 12.0%	5/53 9.4%
First degree relative with diabetes	13/50 26.0%	6/53 11.3%
24–28 weeks	*n* = 50	*n* = 53
Weight gain from pre-pregnancy	**6.7 ± 5.9 (*n* = 49)**	4.1 ± 5.0 (*n* = 46) *
Fasting Blood Glucose (BG)	4.59 ± 0.40	4.57 ± 0.43
1-h BG	7.86 ± 1.64	7.93 ± 1.60
2-h BG	6.23 ± 1.31	6.09 ± 1.20
HOMA-Insulin Resistance	2.86 (2.18, 3.82)	2.54 (2.16, 3.02)
HOMA-Insulin secretion	284 (203, 394)	249 (198, 352)
Stumvoll phase 1	1762 (1386, 2371)	1943 (1311, 2327)
Stumvoll phase 2	455 (359, 606)	501 (346, 594)
GDM at 24–28 weeks	9/49 (18.4%)	9/45 (20.0%)
35–37 weeks	*N* = 46	*N* = 42
Weight gain from pre-pregnancy †	**11.3 ± 6.7 (*n* = 40)**	7.0 ± 6.0 (*n* = 36) *
FBG &	4.53 ± 0.46	4.49 ± 0.51
1-h BG &	8.57 ± 1.31	8.32 ± 1.44
2-h BG &	6.70 ± 1.23	6.59 ± 1.01
HOMA-Insulin Resistance &	2.89 (2.07, 4.44)	2.56 (2.28, 3.84)
HOMA insulin secretion &	309 (233, 504)	337 (242, 411)
Stumvoll phase 1 &	2644 (1793, 3179)	2577 (2001, 3189)
Stumvoll phase 2 &	674 (457, 802)	654 (512, 811)
GDM at 35–37 weeks	9/39(23.1%)	5/36 (13.9%)
Birth outcomes	*N* = 45	*N* = 47
Gestational age at birth	39.8 ± 1.5	39.8 ±1.2
LGA	**7/42 16.7%**	2/44 4.5% *
SGA	1/42 2.4%	4/44 9.1%
Birthweight	3588 ± 524	3455 ± 463
Preterm birth	2/44 4.5%	0/45 0%
Caesarean section	14/43 30.2%	14/47 29.8%
Pre-eclampsia	4/42 9.5%	2/43 4.7%
Birthweight ≥4 kg	9/45 20.0%	7/45 15.6%
Birthweight <2.5 kg	1/45 2.2%	1/45 2.2%
NICU admission	7/41 17.1%	6/44 13.6%
GDM total	15/46 32.6%	13/44 29.5%

Numbers in bold highlight statistically significant comparisons. The HE and PA comparison alone is reported, as the intervention group used for selecting the sites, and the intervention with a significant effect on GWG limitation. There was only one woman with previous GDM in the HE and PA group. All: mean ± SD or *n* (%). The differences between the groups at baseline were tested using *T*-test or Mann–Whitney U-test, as appropriate. For the continuous outcomes, the differences between groups at 24–28 and 35–37 weeks were tested in mixed models, adjusted for baseline values, and for the outcome weight gain adjusted for pre-pregnancy BMI. For the dichotomous outcomes, a logistic regression was performed, and was adjusted for country. * *p* < 0.05 ** *p* < 0.01 vs. UC. &—values of 24–28 weeks (T3) carried forward to 35–37 weeks (T4) when GDM was present at T3. &–excluding women with GDM at T3.
